# Ferroportin-Hepcidin Axis in Prepubertal Obese Children with Sufficient Daily Iron Intake

**DOI:** 10.3390/ijerph15102156

**Published:** 2018-10-01

**Authors:** Joanna Gajewska, Jadwiga Ambroszkiewicz, Witold Klemarczyk, Ewa Głąb-Jabłońska, Halina Weker, Magdalena Chełchowska

**Affiliations:** 1 Screening Department and Metabolic Diagnostics, Institute of Mother and Child, Kasprzaka 17a, 01-211 Warsaw, Poland; jadwiga.ambroszkiewicz@imid.med.pl (J.A.); ewa@rglab.pl (E.G.-J.); magdalena.chelchowska@imid.med.pl (M.C.); 2 Department of Nutrition, Institute of Mother and Child, Kasprzaka 17a, 01-211 Warsaw, Poland; witold.klemarczyk@imid.med.pl (W.K.); halina.weker@imid.med.pl (H.W.)

**Keywords:** ferroportin, hepcidin, iron markers, obesity, children

## Abstract

Iron metabolism may be disrupted in obesity, therefore, the present study assessed the iron status, especially ferroportin and hepcidin concentrations, as well as associations between the ferroportin-hepcidin axis and other iron markers in prepubertal obese children. The following were determined: serum ferroportin, hepcidin, ferritin, soluble transferrin receptor (sTfR), iron concentrations and values of hematological parameters as well as the daily dietary intake in 40 obese and 40 normal-weight children. The ferroportin/hepcidin and ferritin/hepcidin ratios were almost two-fold lower in obese children (*p* = 0.001; *p* = 0.026, respectively). Similar iron concentrations (13.2 vs. 15.2 µmol/L, *p* = 0.324), the sTfR/ferritin index (0.033 vs. 0.041, *p* = 0.384) and values of hematological parameters were found in obese and control groups, respectively. Iron daily intake in the obese children examined was consistent with recommendations. In this group, the ferroportin/hepcidin ratio positively correlated with energy intake (*p* = 0.012), dietary iron (*p* = 0.003) and vitamin B_12_ (*p* = 0.024). In the multivariate regression model an association between the ferroportin/hepcidin ratio and the sTfR/ferritin index in obese children (β = 0.399, *p* = 0.017) was found. These associations did not exist in the controls. The results obtained suggest that in obese children with sufficient iron intake, the altered ferroportin-hepcidin axis may occur without signs of iron deficiency or iron deficiency anemia. The role of other micronutrients, besides dietary iron, may also be considered in the iron status of these children.

## 1. Introduction

The ferroportin-hepcidin axis is a system that plays an important role in iron homeostasis regulating the absorption of dietary iron via the enterocytes of the proximal duodenum and releasing stored iron from hepatocytes and reticuloendothelial macrophages [1]. Ferroportin (FPN1), encoded by the *SLC40A1* gene, is a 62.5 kDa protein consisting of 12 transmembrane domains with the N-terminus and the C-terminus located intracellularly [2]. FPN1 is expressed by the enterocytes of the duodenum, macrophages, and hepatocytes and is responsible for the release of iron to transferrin. However, the mechanism of iron transport by FPN1 is not well known. This protein is the sole cellular iron exporter regulated through posttranslational mechanisms by hepcidin and iron transport [3,4]. Moreover, FPN1 is the only known receptor for hepcidin, a cysteine-rich peptide, whose gene is located on chromosome 19 in humans. Inactivation of this gene was associated with severe iron overload in the liver and the pancreas [5].

The hepcidin gene encodes a precursor prepropeptide of 84 amino acids. Next this precursor is processed by two sequential cleavages to produce a mature peptide of 25 amino acids [6]. Hepcidin-25 binds an extracellular loop of FPN involving disulfide bridging, ferroportin ubiquitination and proteasomal degradation. The degradation of the hepcidin/FPN1 complex induces a down-regulation of FPN1 and consequent intracellular sequestration of iron [7]. Iron deficiency is an important health problem affecting people in both developing and developed countries with a high socio-economic status [8]. This problem particularly concerns two main at-risk target groups: preschool children and young women [9]. The iron deficiency may affect school children (above 5 years) to a lesser extent than it does preschool children, but due to the increased demand related to the growth and development of the body, school children may also be at risk of iron deficiency and iron deficiency anemia [10]. In addition, obesity-related iron deficiency may impair brain development in children, which may disturb cognitive function, motor skills and behavior [11]. Therefore, prevention of iron deficiency is important in school children, especially among those of them who are starting their education [12]. Demand for iron is the highest in the first 6 months of life; up to the age of 3 years it is at a slightly lower level, and in the subsequent years of life it grows, gradually reaching maximum values in adolescence [11]. In overweight/obese children, iron deficiency was a frequent finding but the mechanism for diminished iron status in these subjects is not known. One of the proposed causes of hypoferritinemia in obesity is deficient iron intake from an iron poor diet [13], but some studies suggest this is not the cause [14,15]. Several researchers have demonstrated that iron deficiency is associated with the higher circulation of hepcidin in obese subjects, including children and adolescents [16,17,18,19]. Other studies showed lower values of hepcidin-25 and hyperferritinemia in overweight and obese children compared with their normal-weight counterparts [20]. Changes in hepcidin expression may be mediated by inflammatory response, which is suggested as another mechanism for obesity-related low iron levels [21]. Obesity is characterized by low-grade systemic inflammation with increased levels of C-reactive protein (CRP), proinflammatory cytokines and adipose-derived cytokines, including leptin and adiponectin which may play a role in iron homeostasis [18]. The changes in hepcidin concentrations may be mediated by iron therapy, which was observed in non-obese children with iron deficiency anemia [22]. In turn, some authors reported that iron deficiency observed in obesity was independent of hepcidin expression but may depend on iron absorption-related duodenal enzymes [23]. Studies concerning hepcidin levels in healthy pediatric populations are limited [24,25,26]. However, the significant correlation between hepcidin and ferritin levels observed by the authors may suggest the usefulness of hepcidin as a marker in detecting various iron-related disorders as well as subclinical iron deficiency [26,27].

It is suggested that hepcidin downregulates ferroportin expression and dominantly controls the iron export activity of ferroportin in patients with cancer, Alzheimer’s disease, and anemia of chronic inflammation [28,29,30]. Therefore, the ferroportin-hepcidin axis may play a role as a therapeutic target in anemia and iron overload disorders [5]. So far, the role of the ferroportin-hepcidin axis in the pathophysiology of iron metabolism in obesity has not been examined. The aim of this study was to assess the iron status, especially ferroportin and hepcidin concentrations, in prepubertal obese children. Furthermore, the associations between the ferroportin-hepcidin axis and other iron status markers were analyzed.

## 2. Materials and Methods

### 2.1. Patients

Eighty prepubertal children aged 5–9 years were recruited from a group of consecutive patients (125 children) seeking dietary counseling in the Department of Nutrition at the Institute of Mother and Child in Warsaw. The obese group consisted of 40 children chosen according to the following inclusion criteria: (a) school age (above 5 years), (b) prepubertal period, (c) (body mass index (BMI Z-score > 2) (WHO Reference Curves, The ECOG’s eBook on Child and Adolescent Obesity, 2015). The exclusion criteria were: (a) pubertal exam; (b) the presence of endocrine disorders or genetic syndromes, including syndromic obesity; (c) acute or chronic medical conditions; (d) intake of medications that could affect growth, pubertal development, nutritional or dietary status; (e) not signing the informed consent form. The control group consisted of 40 prepubertal non-obese children (BMI Z-score (−1 + 1)) within the same age range as the obese group (there is a tendency for age differences to result from examining a slightly younger control group coming for dietary counseling) with an adequate nutritional or dietary status according to the recommendations of Kułaga et al. [31] and Jarosz et al. [32]. These children were consulted due to their parents’ opinion that they had a reduced appetite and ate too little. The control group in the study were children: (a) without either acute or chronic disorders (b) not taking any medications that could affect their development and nutritional or dietary status (c) whose parents did not sign the informed consent form. All of the participants were Caucasian. Pubertal stage was determined according to the Tanner scale by the physician during the medical appointment at which the patients were enrolled in either the obese or non-obese group. Written informed consent was obtained from the parents of all the children who were examined. The study was performed in accordance with the Helsinki Declaration for Human Research, and the study protocol was approved (No. 9/18) by the Ethics Committee of the Institute of Mother and Child in Warsaw, Poland.

### 2.2. Assessment of Dietary Intake

Two weeks before the child was due to visit the Department of Nutrition, a 14-day food diary was completed at home using a questionnaire and brought to the Institute. The parents had previously been trained by a nutritionist to provide reliable estimates of diary intake. Three parents prepared 10-day food diaries; they omitted 4 days. In the Nutrition Department, nutritionists carried out an interview concerning the family and environmental conditions of the children, their nutritional behaviors and food preferences, and checked the diary in the presence of the child and his/her parents. The nutritionist asked for detailed information about the foods and drinks recorded, such as portion sizes and preparation methods. The serving sizes were estimated and when necessary the portion sizes were corrected during the visit. This was done by the nutritionist on the basis of an interview with the parents using a photo album of products and dishes presenting meal portion sizes [33]. The 3-consecutive-day methodology was used according to the methodological guide on nutrition research to assess the intake in the children’s dietary habits [34]. The data of the 3-day dietary records, on three consecutive days (two weekdays and one weekend day), were entered into nutritional analysis software (Dieta 5^®^, National Food and Nutrition Institute, Warsaw, Poland) to evaluate the average daily energy intake and the percentage of energy intake from protein, fat and carbohydrates as well as dietary mineral and vitamin (iron, vitamin C, B_12_) intakes in the children’s diets [35]. The data for each child were compared to the recommendations for appropriate age and gender. The age- and sex-specific percentage of Estimated Energy Requirement (EER) for total energy intake and percentage of Estimated Average Requirement (EAR) for iron, vitamin C and B_12_ were calculated. Since the children studied are between 5 and 9 years old, they fall into either one of the two groups (4–6 years or 7–9 years) or one group (4–18 years), according to the recommendations of Jarosz et al. [32]. The participants in the present study did not receive supplements except the standard supplementation with vitamin D.

### 2.3. Anthropometric Measurements

Physical examinations, including body height and weight measurements, were performed in both of the studied groups. Body height was measured using a standing stadiometer and recorded with a precision of 1 mm. Body weight was assessed unclothed, to the nearest 0.1 kg, with a calibrated balance scale. The Body Mass Index (BMI) was calculated as body weight divided by height squared (kg/m^2^). The BMI of each individual was converted to BMI Z-score for the child’s age and sex using Polish reference tables [31]. The data of this reference population were derived from a study concerning the physical development of Polish children and adolescents from birth to 18 years of age.

### 2.4. Biochemical Analyses

Venous blood samples were collected between 8:00 and 10:00 a.m. after an overnight fast. Blood in EDTA-containing tubes was analyzed immediately for the determination of hemoglobin (Hb), red blood cells (RBC), and mean corpuscular volume (MCV) using a hematology analyzer (Horiba ABX, Montpellier, France). To obtain serum, the blood was centrifuged at 1000 × g for 10 min at 4 °C. Serum specimens were stored at −70 °C prior to assay. Serum iron, ferritin and CRP as an inflammatory marker were analyzed using commercially available kits on a biochemical analyzer (Roche, Basel, Switzerland). Serum ferroportin, hepcidin, soluble transferring receptor (sTfR), leptin and proinsulin concentrations were determined by immunoenzymatic methods. Serum ferroportin was measured using the Human FPN ELISA kit (Elabscience, Houston, TX, USA) with anti-human FPN antibody, which had intra- and inter-assay CVs of less than 5.4% and 6.1%, respectively. Serum hepcidin and soluble transferrin receptor (sTfR) concentrations were analyzed using Elisa kits (DRG, Marburg, Germany) with anti-human hepcidin-25 (bioactive hepcidin molecule) or anti-human sTfR antibodies. The intra- and inter-assay CVs were less than 5.7% and 9.5% for hepcidin and 6.0% and 7.0% for sTfR, respectively. Elisa kits from DRG (Germany) and TECO (Sissach, Switzerland) were used to determine leptin and proinsulin concentrations, respectively. The intra- and inter–assay CVs were less than 7.3% and 9.1% for leptin and 2.2% and 4.0% for proinsulin, respectively. The analysis of each parameter was performed in duplicate.

### 2.5. Statistical Analyses

Statistical analysis was performed using STATISTICA 10.0 (StatSoft Inc., Tulsa, OK, USA). The results are presented as means ± standard deviation (SD) for normally distributed data or medians and interquartile range (25th–75th percentiles) for non-normally distributed variables as well as minimum and maximum values. The Kolmogorov-Smirnov test was used to evaluate distribution for normality. Differences in anthropometric characteristics, dietary intake, and biochemical parameters of obese and non-obese children were assessed using the Student’s *t*-test test for normally distributed data and the non-parametric Mann-Whitney *U* test for non-normally distributed variables. The age- and sex-matched groups were compared using the exact Wilcoxon test. Pearson or Spearman correlations between parameters of FPN1, hepcidin, as well as the ferroportin/hepcidin ratio and anthropometric, biochemical and dietary parameters were calculated. The multivariate regression model with the ferroportin/hepcidin ratio as a dependent variable was assessed to examine the potential impact of the anthropometric, dietary, and biochemical variables. The results were presented as the value of β standardized regression coefficient and a change in R-squared coefficient of determination after each variable was entered. Differences were regarded as statistically significant at *p* < 0.05. 

## 3. Results

Clinical, anthropometric, hematologic, and biochemical characteristics of the study participants are shown in Table 1. As expected, height, weight, and BMI were significantly higher in obese than in non-obese children of the same age. The children with obesity had elevated serum leptin and proinsulin (*p* < 0.001) concentrations. Serum hepcidin concentration was increased by about 40% (*p* < 0.018) but serum FPN1 concentration was reduced by 30% (*p* < 0.040) compared with the normal-weight controls. The ferroportin/hepcidin (*p* = 0.001) and ferritin/hepcidin (*p* = 0.026) ratios were almost two-fold lower in obese children than in the controls. However, similar values of the sTfR/ferritin index and serum levels of sTfR, ferritin and iron were found in both of the studied groups. In obese children, the hematological parameters, such as hemoglobin, RBC and MCV were within the reference range and similar to the normal-weight group.

The median values of the inflammation marker, CRP was significantly higher (*p* < 0.001) in the obese group compared with the normal-weight group, but CRP levels were within the normal range (below 5 mg/L) in all the individuals. Analyzing the age- and sex-matched groups, a similar tendency was observed between the 29 obese and non-obese children (Table 2). 

Significant differences were found for leptin (<0.001), proinsulin (*p* < 0.001), ferroportin (*p* = 0.023), hepcidin (*p* = 0.002), CRP (*p* < 0.001) concentrations, and ferroportin/hepcidin (*p* < 0.001), as well as ferritin/hepcidin (*p* = 0.002) ratios. Other biochemical parameters were similar in both of the studied groups. 

The daily energy intake in children with obesity was higher (*p* < 0.001) compared with the controls, but the proportions of proteins, carbohydrates, and fats in daily energy intake were similar in both groups (*p* > 0.05), and consistent with recommendations concerning daily intake for children aged 4–18 years (Table 3).

The percentage of EER was significantly higher in obese than in non-obese children (*p* < 0.001). The diet of obese children contained a higher intake of iron (*p* = 0.005) and vitamin C (*p* = 0.010) than that of normal-weight children. The percentage of EAR for iron and vitamin C intakes were also significantly higher in obese than in non-obese children (*p* < 0.010; *p* < 0.025, respectively), (EAR for children aged 4–9 years, iron: 4 mg/day, vitamin C: 40 mg/day). A similar intake of vitamin B_12_ was observed in obese and normal-weight children and consistent with the recommendations concerning daily intake. Analyzing the age- and sex-matched groups, a similar tendency concerning dietary intake was observed in 29 obese and non-obese children (Table 4). Significant differences were found in energy intake (*p* < 0.001), percentage of EER (*p* < 0.001), iron intake (*p* = 0.011), and vitamin C intake (*p* = 0.04). The intake of other nutrients was similar in both of the studied groups.

In correlation analyses defined for both of the studied groups a negative relation between FPN1 levels and dietary iron (*p* = 0.013), vitamin C (*p* = 0.011) and vitamin B_12_ (*p* = 0.033) were found in the control group (Table 5). No relations between FPN1 levels and anthropometrical, biochemical and dietary parameters in the obese group were observed.

As shown in Table 6, hepcidin concentrations negatively correlated with BMI (*p* = 0.022), leptin (*p* = 0.006), and the sTfR/ferritin index (*p* = 0.008) and positively with ferritin concentrations (*p* = 0.009) in obese children. In normal-weight children, relations between hepcidin levels and the percentage of dietary proteins (*p* = 0.015) and sTfR levels were found (*p* = 0.011).

In the obese group, the ferroportin/hepcidin ratio correlated positively with energy intake (*p* = 0.012), dietary iron (*p* = 0.003) and vitamin B_12_ (*p* = 0.024) (Table 7). It was also observed that this ratio positively correlated with the sTfR/ferritin index (*p* < 0.001) and negatively with ferritin levels (*p* = 0.002). These correlations were more significant with the ferroportin/hepcidin ratio than with separate hepcidin or ferroportin levels (Table 3 and Table 4). In normal-weight children, no relations between the ferroportin/hepcidin ratio and anthropometric, biochemical, and dietary parameters in the obese group were observed.

In the multivariate regression model, an association between the ferroportin/hepcidin ratio and the sTfR/ferritin index was found (β = 0.399, *p* = 0.017) in the obese group, but no such correlation was observed in normal-weight children (Table 8).

R-squared (expressed as a percentage of a variation that can be explained by a linear regression model) was 46.9% for obese children and 20.8% for the controls. An effect of collinearity that could affect the results was not found.

## 4. Discussion

Some studies showed that iron deficiency was a frequent finding in obese children [14,16], adolescents [18] and adults [15,36] but others found hyperferritinemia and a lower sTfR/ferritin index in these subjects [37,38]. The present study found normal serum levels of iron, ferritin, sTfR and the sTfR/ferritin index, despite a reduced ratio of ferroportin to hepcidin in prepubertal obese children with sufficient daily iron intake. In addition, the results obtained from the relatively small obese group that was examined did not indicate the occurrence of iron deficiency and iron deficiency anemia in these subjects.

Higher values of hepcidin were observed in obese children and adolescents by many authors [14,16,17,18,39], although Chang [20] found lower values of hepcidin in these subjects compared with the controls. In addition, some authors showed that higher concentrations of hepcidin were associated with lower iron and ferritin values and higher concentrations of sTfR in obese children [14,17,18]. These authors suggest that hypoferritinemia and elevated hepcidin are prevalent in obese children and hepcidin is an important modulator of anemia in obesity. 

In this study similar concentrations of ferritin and iron were found in obese and non-obese groups. This is similar to the results obtained by Aeberli et al. [14], Sanad et al. [16] and Sal et al. [19]. Ferritin is considered an indicator for determining iron deficiency, but serum ferritin levels had weak correlations with serum iron levels and transferrin saturation [40]. Hence, it is suggested that both serum ferritin and sTfR should be used as better markers than ferritin alone for measuring the iron status of populations. STfR is less affected by the acute phase response and may be more useful when assessing iron metabolism in chronic diseases with inflammatory status [21]. The obese children examined in the study presented had significantly higher concentrations of CRP than the non-obese group, although these values were still within the normal range. In addition, higher values of leptin in obese than non-obese subjects were found. This may confirm that pediatric obesity can be considered a low-grade inflammatory state that stimulates the production of inflammatory factors.

The same sTfR concentrations and sTfR/ferritin index were observed in the obese group and the controls, which suggests a similar iron status in both of the studied groups. Unlike the results obtained in the present study, Aeberli et al. [14] and Nazif et al. [18] found higher concentrations of sTfR associated with higher values of hepcidin and CRP in obese children and adolescents. According to these authors, iron deficiency is related to hepcidin-mediated reduced iron absorption and/or increased iron sequestration. However, Sal et al. [19] observed no significant differences in terms of hemoglobin, serum ferritin, and iron in obese children aged from 5 to 18 years. Despite a higher value of hepcidin, the authors suggest that this parameter does not contribute to the development of iron deficiency in obese children. In the present study similar values of iron markers and hematological parameters, such as hemoglobin, RBC and MCV in obese children, were found together with lower values of the ferroportin/hepcidin ratio.

A lower value of the ferroportin/hepcidin ratio due to lower ferroportin and higher hepcidin concentrations were observed in obese patients than in normal-weight children. De Domenico et al. [41] suggested that hepcidin regulates FPN1-mediated iron export by regulating the concentration of FPN1 rather than the transport activity of FPN1. Hepcidin production by the liver is simultaneously regulated by different factors, including: iron status, hypoxia, and inflammation [42,43]. According to Zhao and Enns [44] and Becker et al. [45], the down-regulation of FPN1 results in reduced iron export across the enterocyte membrane and increased iron sequestration within intestinal enterocytes, hepatocytes and iron-recycling macrophages causing local iron overload. Many studies in obese subjects demonstrated increased iron content in the liver, macrophages, and adipose tissue producing the condition for the adverse effect of iron overload [5,46].

It is known that reticulo-endothelial macrophages together with duodenal enterocytes coordinate body iron homeostasis, but there are data concerning differential response of ferroportin-expressing cells to hepcidin action. According to Chaston [47], the primary targets of hepcidin are iron-recycling macrophages present in the spleen, rather than enterocytes. Therefore, the duodenum appears to be less sensitive to this initial rise in hepcidin levels. The fact that macrophages respond more acutely to a hepcidin challenge is consistent with their central role in maintaining body iron homeostasis [48]. Some studies have suggested that adiposity predicts decreased intestinal iron absorption [15,49] but in the present study relations between either the ferroportin/hepcidin ratio and BMI or leptin concentrations in obese children were not observed. According to Zimmerman et al. [15], the contribution of BMI to a reduction in iron absorption seems to be low. 

Obesity may be associated with diets low in iron, therefore iron-poor diets were proposed to be responsible for the disturbances in plasma iron concentrations [50]. However, when dietary iron intakes in obese subjects are estimated, they are not lower than in normal weight individuals [51,52]. Total iron consumption is often similar in obese and non-obese children and adults, whereas heme iron intake and the consumption of animal protein is higher in obese subjects [14]. Aeberli et al. [14] also reported no significant differences in dietary iron intake or bioavailability in 6–14-year-old obese and non-obese children, but higher hepcidin concentration and poorer iron status in comparison with the control group were found. Although it is indicated that obese children may risk having micronutrient deficiencies, including that of vitamin C [53], the present study showed higher daily intake of iron as well as vitamin C in obese children than in the controls. In obese children and adolescents similar or higher values of vitamin C intake in comparison to those reported by other authors were found [13]. It is known that this vitamin acts by reducing Fe^3+^ to the more soluble Fe^2+^ and improves dietary iron bioavailability. In addition, vitamin C reduces the inhibitory effect of phytates and polyphenols on mineral absorption. Therefore, higher intake of vitamin C in the diet could partly have counteracted the inhibitory effect of these compounds’ intake on nonheme iron absorption in the obese children examined in this study. Moreover, a positive relation between total energy, dietary iron, vitamin B_12_, and the ferroportin/hepcidin ratio were observed. This may suggest that besides dietary iron, other micronutrients, such as vitamins may influence the iron status of these obese children. 

In the study presented, lower values of the ferroportin/hepcidin ratio were found in obese than in non-obese children and positive relations between this ratio and the sTfR/ferritin index in the obese group were slightly more significant than when ferroportin and hepcidin concentrations were considered separately. However, the study group is relatively small and the control group was slightly younger than the obese group. Additionally, pediatric studies defining reference ranges for hepcidin and especially ferroportin are limited [25,26] but some studies do consider age differences in hepcidin levels in healthy children. [25,54,55]. Uijterschout et al. [54] observed differences in hepcidin levels in Dutch children up to 3 years of age. Aranda et al. [55] found that hepcidin levels increased in healthy Spanish infants during the first year of life. Higher values of this parameter were observed in older Greek children (10–12 years) than in younger ones by Sdogou et al. [25]. Since the influence of age on the level of the studied parameters which related to iron metabolism cannot be excluded, further research concerning the relations between the ferroportin/hepcidin axis in obesity are needed and should be conducted on a larger group of subjects, assessing the potential impact of age on the hepcidin as well as the ferroportin levels.

The present study has some limitations. Firstly, the findings were obtained from a relatively small sample of subjects. In addition, analyzing the age- and sex-matched groups, the entire obese and control groups should be included. Both groups were homogenous in terms of the prepubertal period and were characterized anthropometrically and metabolically. Although the difference in age between the groups was not statistically significant, there is a tendency for differences to result from examination a slightly younger control group than the obese group described here. The second limitation is its cross-sectional nature and the absence of a prospective longitudinal analysis. Therefore, long-term observations, including weight-loss therapy, are needed to examine the relationship between the markers of iron status and the clinical outcomes in the subjects. Thirdly, the consecutive 3-day dietary record instead of generally applied non-consecutive ones was used for the assessment of dietary intake. However, the consecutive 3-day method was described in the methodological guide on nutrition research [34] and is still used in nutritional analyses by other authors [56,57,58]. For confirmation of the results obtained, however, using the non-consecutive method on a larger study group of children is advisable, particularly for the micronutrient evaluation. The next limitation is the assessment of FPN1 concentrations in the obese and non-obese groups. There are two main isoforms of ferroportin, FPNA1 and FPNB1, differing e.g., in the localization in various tissues and organs [59]. FPNB1 is expressed mainly in duodenal enterocytes and may export iron to the organism even if the enterocytes become iron deficient. The role of both of these ferroportin forms in relations between the ferroportin-hepcidin axis and other iron markers in obesity require further study. Moreover, other isoforms of hepcidin-25 have been identified (hepcidin-20, -22, and -24), but these forms probably do not influence iron transport [60].

## 5. Conclusions

The results obtained in the study presented suggest that in obese children with sufficient iron intake, the altered ferroportin-hepcidin axis may occur without signs of iron deficiency or iron deficiency anemia. The role of other micronutrients, besides dietary iron, may also be considered in the iron status of these children.

## Figures and Tables

**Table 1:** Clinical and biochemical characteristics in prepubertal children with obesity and in normal-weight children.

Parameter	Children with Obesity *n* = 40	Normal-Weight Children *n* = 40	*p*-Value
Age (years)	7.8 (6.5–8.6)	6.5 (5.3–8.7)	0.062
	{5.0–9.9}	{5.0–9.9}	
Male (%)	50.0	57.5	0.753
**Anthropometric parameters**			
Height (cm)	135.7 (122.9–140.0)	116.0 (110–123.8)	<0.001
	{116.0–151.0}	{105.0–148.0)	
Weight (kg)	42.3 (36.0–51.4)	18.8 (17.4–22.0)	<0.001
	{26.3–64.8}	{15.4–40.0}	
BMI (kg/m^2^)	23.7 (21.7–25.3)	14.8 (14.2–16.0)	<0.001
	{19.2–30.3}	{13.7–18.5}	
BMI Z-score	3.14 ± 1.2	−0.52 ± 0.45	<0.001
	{1.99–5.99}	{−1.08–0.35}	
**Biochemical measurements**			
Leptin (ng/mL)	9.65 (5.97–17.15)	1.71 (1.17–2.89)	<0.001
	{2.59–44.27}	{0.48–4.77}	
Proinsulin (pmol/L)	2.76 (1.76–4.05)	0.21 (0.08–1.18)	<0.001
	{0.97–5.84}	{0.02–3.61}	
Ferroportin (ng/mL)	16.5 (12.9–20.1)	23.5 (14.5–30.5)	0.040
	{9.2–42.0}	{7.2–48.0}	
Hepcidin (ng/mL)	15.3 (8.7–21.3)	11.1 (6.8–14.5)	0.018
	{5.9–54.5}	{2.4–37.1}.	
Ferroportin/Hepcidin	1.12 (0.65–1.97)	2.13 (1.38–3.49)	0.001
	{0.18–3.77}	{0.25–4.08}	
sTfR (mg/L)	1.03 (0.84–1.25)	1.03 (0.93–1.28)	0.564
	{0.65–1.86}	{0.56–2.03}	
Ferritin (ng/mL)	29.5 (20.0–44.0)	25.7 (21.0–41.4)	0.531
	{12.1–70.0}	{14.4–59.0}	
sTfR/Ferritin	0.033 (0.023–0.055)	0.041 (0.028–0.056)	0.384
	{0.016–0.103}	{0.012–0.117}	
Ferritin/Hepcidin	2.06 (1.44–2,86)	3.17 (1.76–5.29)	0.026
	{0.34–5.69}	{0.63–6.99}	
Iron (µmol/L)	13.2 (10.8–19.8)	15.2 (11.9–17.4)	0.324
	{6.3–32.3}	{6.8–32.6}	
RBC (×10^6^/µL)	4.81 ± 0.26	4.69 ± 0.22	0.050
	{4.21–5.49}	{3.96–5.12}	
Hb (g/dL)	12.86 ± 1.01	13.00 ± 0.55	0.734
	{11.0–14.3}	{11.9–14.6)	
MCV (fL)	81.20 ± 2.77	82.71 ± 2.84	0.117
	{76.9–87.0}	{77.0–86.0)	
CRP (mg/L)	1.40 (0.75–3.07)	0.22 (0.09–0.56)	<0.001
	{0.01–5.05}	{0.04–4.20}	

The results are presented as means ± standard deviations for normally distributed data or medians and interquartile ranges (25th–75th percentiles) for non-normally distributed variables, and as {minimum-maximum}, BMI—body mass index, sTfR—soluble transferrin receptor, RBC—red blood cells, Hb –hemoglobin, MCV—mean corpuscular volume, CRP—C-reactive protein.

**Table 2:** Clinical and biochemical characteristics in prepubertal children with obesity and in normal-weight children (pair-matched group).

Parameter	Children with Obesity *n* = 29	Normal-Weight Children *n* = 29	*p*-Value
Age (years)	7.5 (6.2–8.8)	7.5 (6.1–8.9)	0.807
	{5.0–9.9}	{5.0–9.9}	
Male (%)	52.2	52.2	
**Anthropometric parameters**			
Height (cm)	134.5 (122.5–145.3)	119.0 (112–127)	<0.001
	{116.0–151.0}	{105.0–148.0)	
Weight (kg)	41.1 (34.9–55.2)	20.0 (17.7–23.5)	<0.001
	{26.3–64.8}	{15.4–40.0}	
BMI (kg/m^2^)	23.2 (22.1–25.5)	14.8 (14.2–16.4)	<0.001
	{19.2 -30.3}	{13.7–18.5}	
BMI Z-score	3.38 ± 1.24	−0.44 ± 0.47	<0.001
	{1.99–5.99}	{−1.08–0.35}	
**Biochemical measurements**			
Leptin (ng/mL)	9.51 (5.83–20.44)	1.72 (0.89–3.09)	<0.001
	{2.59–44.27}	{0.48–4.77}	
Proinsulin (pmol/L)	2.74 (1.92–3.50)	1.00 (0.09–1.36)	<0.001
	{1.13–5.66}	{0.04–3.61}	
Ferroportin (ng/mL)	16.4 (11.1–20.6)	25.0 (14.9–29.4)	0.023
	{9.2–42.0}	{7.2–48.0}	
Hepcidin (ng/mL)	17.5 (12.2–25.3)	10.1 (6.3–12.9)	0.002
	{5.9–54.5}	{3.1–37.0}	
Ferroportin/Hepcidin	0.97 (0.53–1.85)	2.41 (1.48–3.83)	<0.001
	{0.18–3.77}	{0.25–4.08}	
sTfR (mg/L)	1.03 (0.87–1.22)	1.02 (0.92–1.41)	0.498
	{0.65–1.54}	{0.56–2.03}	
Ferritin (ng/mL)	32.0 (22.0–45.0)	26.0 (20.5–48.0)	0.555
	{12.1–63.0}	{14.4–58.1}	
sTfR/Ferritin	0.031 (0.022–0.055)	0.039 (0.022–0.059)	0.518
	{0.016–0.077}	{0.012–0.099}	
Ferritin/Hepcidin	1.86 (1.39–2.82)	4.00 (1.75–5.29)	0.002
	{0.34–4.95}	{0.63–6.99}	
Iron (µmol/L)	14.0 (11.0–19.4)	15.3 (12.1–17.8)	0.665
	{6.3–32.3}	{6.8–26.1}	
RBC (×10^6^/µL)	4.85 ± 0.35	4.79 ± 0.29	0.057
	{4.31–5.49}	{3.96–5.12}	
Hb (g/dL)	12.9 ± 0.92	13.00 ± 0.57	0.544
	{11.0–14.3}	{11.9–13.9)	
MCV (fL)	80.30 ± 2.57	82.38 ± 2.74	0.056
	{76.9–86.0}	{77.0–86.0)	
CRP (mg/L)	1.36 (0.71–3.76)	0.28 (0.08–0.78)	<0.001
	{0.01–5.05}	{0.06–4.20}	

The results are presented as means ± standard deviations for normally distributed data or medians and interquartile ranges (25th–75th percentiles) for non-normally distributed variables, and as {minimum-maximum}, BMI—body mass index, sTfR—soluble transferrin receptor, RBC—red blood cells, Hb—hemoglobin, MCV—mean corpuscular volume, CRP—C-reactive protein.

**Table 3:** Daily energy and nutrient intake of the examined children compared with recommended daily intake.

Parameter	Children with Obesity *n* = 40	Normal-Weight Children *n* = 40	*p*-Value	EER or EAR * (Years, Intake)
Energy (kcal/24h)	1742 (1569–1923)	1500 (1254–1580)	<0.001	4–6 years, 1400; 7–9 years, 1800
	{1290–3905}	{1180–2157}		
Energy (% of EER)	99.2 (92.4–115.9)	86.3 (78.5–103.2)	<0.001	
	{73.3–216.9}	{66.7–119.8}		
Proteins (% of energy intake)	14.5 ± 2.4	13.6 ± 1.9	0.147	4–18 years, 10–20
	{9.6–21.2}	{10.0–19.6}		
Carbohydrates (% of energy intake)	53.5 ± 6.1	53.2 ± 7.6	0.201	4–18 years, 45–65
	{34.6–62.2}	{30.4–60.8}		
Fat (% of energy intake)	32.0 ± 5.9	32.0 ± 6.3	0.724	4–18 years, 20–35
	{21.7–46.2}	{19.5–39.3}		
Iron (mg/day)	9.00 (7.84–10.20)	7.70 (6.90–8.90)	0.005	4–6 years, 4; 7–9 years, 4
	{5.07–19.1}	{6.1–12.4}		
Iron (% of EAR)	225.0 (195.9–255.0)	192.5 (172.5–221.2)	0.010	
	{126.8–477.5}	{152.5–310.0}		
Vitamin C (mg/day)	86.5 ± 45.5	65.7 ± 24.0	0.010	4–6 years, 40; 7–9 years, 40
	{29.2–200.0}	{29.4–126.3}		
Vitamin C (% of EAR)	199.8 (135.6–246.6)	151.4 (120.3–190.0)	0.025	
	{73.0–500.0}	{73.5–315.8}		
Vitamin B_12_ (µg/day)	2.22 (1.65–2.96)	1.80 (1.70–2.10)	0.134	4–6 years, 1.0; 7–9 years, 1.5
	{1.01–7.20}	{1.00–7.05}		
Vitamin B_12_ (% of EAR)	184.0 (115.3–223.4)	160.0 (130.8–197.5)	0.694	
	{67.3–480.0}	{90.0–470.0}		

The results are presented as means ± standard deviations for normally distributed data or medians and interquartile ranges (25th–75th percentiles) for non-normally distributed variables, and as {minimum-maximum} % of EER—percentage of Estimated Energy Requirement, % of EAR—percentage of Estimated Average Requirement. * The data are presented as recommended daily energy and nutrient intake according to Jarosz et al. [32].

**Table 4:** Daily energy and nutrient intake of the examined children (pair-matched group) compared with recommended daily intake.

Parameter	Children with Obesity *n* = 29	Normal-Weight Children *n* = 29	*p*-Value	EER or EAR * (Years, Intake)
Energy (kcal/24 h)	1810 (1615–1960)	1400 (1250–1542)	<0.001	4–6 years, 1400; 7–9 years, 1800
	{1395–2500}	{1180–2157}		
Energy (% of EER)	106.9 (100.0–135.3)	86.3 (75.9–105.4)	<0.001	
	{77.5–170.7}	{66.7–119.8}		
Proteins (% of energy intake)	14.8 ± 2.7	13.6 ± 1.7	0.149	4–18 years, 10–20
	{10.5–21.2}	{10.0–16.0}		
Carbohydrates (% of energy intake)	52.3 ± 6.5	53.0 ± 6.1	0.565	4–18 years, 45–65
	{34.6–61.9}	{40.3–60.8}		
Fat (% of energy intake)	32.8 ± 5.9	32.0 ± 4.9	0.638	4–18 years, 20–35
	{26.1–46.2}	{25.1–39.3}		
Iron (mg/day)	9.30 (8.26–10.30)	7.50 (6.90–9.00)	0.011	4–6 years, 4; 7–9 years, 4
	{6.00–15.5}	{6.1–10.0}		
Iron (% of EAR)	232.5 (206.5–257.5)	187.5 (172.5–225.0)	0.010	
	{150.0–387.5}	{152.5–250.0}		
Vitamin C (mg/day)	79.1 ± 37.4	63.0 ± 23.2	0.040	4–6 years, 40; 7–9 years, 40
	{34.0–200.0}	{29.4–126.3}		
Vitamin C (% of EAR)	195.8 (125.6–236.7)	145.4 (119.3–189.0)	0.028	
	{85.0–500.0}	{73.5–315.8}		
Vitamin B_12_ (µg/day)	2.36 (1.65–3.00)	1.80 (1.70–2.00)	0.060	4–6 years, 1.0; 7–9 years, 1.5
	{1.11–3.79}	{1.30–4.30}		
Vitamin B_12_ (% of EAR)	174.0 (125.3–223.4)	158.1 (129.8–187.4)	0.119	
	{80.3–430.0}	{94.0–460.0}		

The results are presented as means ± standard deviations for normally distributed data or medians and interquartile ranges (25th–75th percentiles) for non-normally distributed variables, and as {minimum-maximum}. % of EER—percentage of Estimated Energy Requirement, % of EAR—percentage of Estimated Average Requirement. * The data are presented as recommended daily energy and nutrient intake according to Jarosz at al. [32].

**Table 5:** Correlation analyses of ferroportin with clinical, nutritional data and iron status parameters in the obese and normal-weight groups.

Parameter	Obese	Children	Normal-Weight	Children
	*r*	*p*	*r*	*p*
BMI	−0.229	0.156	−0.074	0.652
Energy	0.067	0.678	−0.243	0.166
Dietary proteins (%)	−0.065	0.691	0.241	0.170
Dietary fat (%)	0.023	0.883	0.006	0.975
Dietary carbohydrates (%)	0.003	0.982	−0.147	0.407
Dietary iron	0.157	0.333	−0.421	0.013
Dietary vitamin C	−0.154	0.343	−0.430	0.011
Dietary vitamin B_12_	0.301	0.059	−0.366	0.033
Leptin	−0.263	0.102	−0.158	0.331
Proinsulin	−0.069	0.133	−0.010	0.540
Hepcidin	−0.040	0.805	0.151	0.353
sTfR	−0.051	0.756	0.068	0.677
Ferritin	0.071	0.664	0.150	0.356
Ferritin/Hepcidin	−0.143	0.379	−0.107	0.511
sTfR/Ferritin	−0.024	0.884	0.049	0.765
Iron	0.088	0.589	0.028	0.862
RBC	−0.206	0.200	−0.017	0.920
Hb	0.044	0.791	0.238	0.142
MCV	0.067	0.680	0.251	0.123
CRP	−0.260	0.105	0.108	0.508

BMI—body mass index, sTfR—soluble transferrin receptor, RBC—red blood cells, Hb—hemoglobin, MCV—mean corpuscular volume, CRP—C-reactive protein.

**Table 6:** Correlation analyses of hepcidin with clinical, nutritional data and iron status parameters in the obese and normal-weight groups.

Parameter	Obese	Children	Normal-Weight	Children
	*r*	*p*	*r*	*p*
BMI	−0.361	0.022	−0.245	0.128
Energy	−0.157	0.334	−0.088	0.619
Dietary proteins (%)	−0.007	0.964	0.414	0.015
Dietary fat (%)	−0.167	0.302	−0.184	0.297
Dietary carbohydrates (%)	0.158	0.329	0.147	0.406
Dietary iron	−0.273	0.088	−0.080	0.651
Dietary vitamin C	−0.121	0.456	−0.193	0.273
Dietary vitamin B_12_	−0.165	0.310	−0.065	0.716
Leptin	−0.424	0.006	0.083	0.609
Proinsulin	−0.308	0.053	−0.219	0.175
Ferroportin	−0.040	0.805	0.151	0.353
sTfR	−0.205	0.205	0.397	0.011
Ferritin	0.410	0.009	−0.098	0.587
sTfR/Ferritin	−0.416	0.008	0.229	0.154
Iron	0.097	0.550	−0.250	0.120
RBC	−0.205	0.202	0.126	0.440
Hb	0.112	0.493	0.044	0.791
MCV	0.102	0.530	−0.120	0.462
CRP	−0.019	0.909	0.129	0.427

BMI—body mass index, sTfR—soluble transferrin receptor, RBC—red blood cells, Hb—hemoglobin, MCV—mean corpuscular volume, RBC—red blood cells, Hb—hemoglobin, MCV—mean corpuscular volume, CRP—C-reactive protein.

**Table 7:** Correlation analyses of the ferroportin/hepcidin ratio with clinical, nutritional data and iron status parameters in the obese and normal-weight groups.

Parameter	Obese	Children	Normal-Weight	Children
	*r*	*p*	*r*	*p*
BMI	0.070	0.669	0.257	0.115
Energy	0.395	0.012	−0.270	0.121
Dietary proteins (%)	−0.266	0.098	−0.271	0.121
Dietary fat (%)	−0.064	0.694	−0.151	0.396
Dietary carbohydrates (%)	0.168	0.301	0.142	0.421
Dietary iron	0.464	0.003	−0.307	0.078
Dietary vitamin C	0.200	0.217	−0.111	0.531
Dietary vitamin B_12_	0.357	0.024	−0.189	0.285
Leptin	0.288	0.071	−0.048	0.770
Proinsulin	0.148	0.363	0.262	0.107
sTfR	0.162	0.317	−0.265	0.103
Ferritin	−0.479	0.002	0.166	0.314
sTfR/Ferritin	0.470	<0.001	−0.191	0.243
Iron	−0.282	0.078	0.091	0.580
RBC	0.050	0.762	−0.018	0.911
Hb	−0.115	0.480	0.153	0.354
MCV	−0.126	0.440	0.168	0.301
CRP	−0.199	0.218	−0.137	0.404

BMI—body mass index, sTfR—soluble transferrin receptor, RBC—red blood cells, Hb—hemoglobin, MCV—mean corpuscular volume, CRP—C-reactive protein.

**Table 8:** Multivariate of the ferroportin/hepcidin ratio with iron status markers, inflammation marker, leptin and some dietary parameters in obese and normal-weight children (adjusted for age, sex, BMI).

Parameter	Obese	Children	Normal-Weight	Children
	β	*p*	β	*p*
Energy	0.177	0.607	−0.211	0.275
Dietary iron	0.262	0.302	−0.231	0.283
Dietary vitamin B_12_	0.081	0.714	−0.094	0.643
Leptin	−0.003	0.987	−0.040	0.845
sTfR/Ferritin	0.399	**0.017**	−0.231	0.220
Iron	−0.122	0.445	0.093	0.612
CRP	−0.140	0.379	−0.084	0.647
*R*^2^ (%)	46.9		20.8	

sTfR—soluble transferrin receptor, CRP—C-reactive protein.
